# The Prevalence of Leakage, Peristomal Skin Complications and Impact on Quality of Life in the First Year Following Stoma Surgery

**DOI:** 10.3390/nursrep15030107

**Published:** 2025-03-19

**Authors:** Richard R. W. Brady, Diane Sheard, Kevin Howard, Martin Vestergaard, Esben Bo Boisen, Rebecca Mather, Rachel Ainsworth, Helle Doré Hansen, Teresa Adeltoft Ajslev

**Affiliations:** 1 Newcastle Centre for Bowel Disease Research Hub, Newcastle Hospitals and Newcastle University, Newcastle upon Tyne NE1 4LP, UK; richard.brady32@nhs.net; 2 Lancashire Teaching Hospital, Royal Preston Hospital, Preston PR2 9HT, UK; diane.sheard@lthtr.nhs.uk; 3 Clinical Trials Research Office, James Paget University Hospital, Great Yarmouth NR31 6LA, UK; kevin.howard@jpaget.nhs.uk; 4 Coloplast A/S, Holtedam 1, 3050 Humlebæk, Denmark; dkmves@coloplast.com (M.V.); dkebb@coloplast.com (E.B.B.); dkhdh@coloplast.com (H.D.H.); 5 Coloplast Ltd., Nene Hall, Peterborough Business Park, Peterborough PE2 6FX, UK; gbrhc@coloplast.com (R.M.);

**Keywords:** stoma, leakage, peristomal skin complications, self-management, quality of life, mental health, nursing

## Abstract

**Objective:** It is well established that having a stoma can negatively impact health-related quality of life (HRQoL), but there is a paucity of research describing the natural history of certain complications associated with living with a stoma, such as leakage and peristomal skin complications (PSCs), and whether these affect QoL within the first year of stoma surgery. The objective of this study was to investigate the pattern of such complications and impact on QoL in individuals who had stoma surgery within the preceding year. **Methods:** A cross-sectional study was conducted at three hospital sites in the United Kingdom to evaluate the burden of disease in those who had undergone intestinal stoma formation surgery within the preceding year. The study consisted of a one-to-one consultation with a study nurse and the completion of an online questionnaire by the patient (ISRCTN-registry: 23080097). The nurse-led interview directly evaluated peristomal skin health, whilst the online questionnaire evaluated the impact of leakage (using the Ostomy Leak Impact tool), generic mental well-being (by WHO-5) and wider HRQoL (by EQ-5D-5L). **Results:** A total of 114 individuals with an intestinal stoma completed the evaluations. The participants had a mean age of 55.8 years (range 18–87 years) and 58% were male. Forty-three percent of the participants had experienced leakage of stomal effluent outside the baseplate (e.g., onto clothes) in the preceding two weeks and 85% suffered from PSCs ranging from mild (35%), to moderate (18%), and severe (32%). Leakage and PSCs were associated with lower mental well-being and HRQoL (*p* < 0.05). Leakage events, HRQoL, mental well-being and peristomal skin health were similar for individuals across different timepoints from the time of surgery within the first year. **Conclusions:** This study reported a high disease burden in people with a new intestinal stoma. Experiencing frequent leakage incidents and/or living with severe PSCs were associated with reduced HRQoL and mental well-being.

## 1. Key Message

The degree to which people struggle with complications associated with living with a stoma and the impact on quality of life and mental well-being is underreported for people in the first year following their stoma formation.

Our study highlights that many people living with a newly formed stoma struggle with complications associated with the stoma, such as leakage, peristomal skin complications, and reduced quality of life and mental well-being. Experiencing leakage and having poor peristomal skin health was associated with poor quality of life and mental well-being. Leakage events, peristomal skin health and QoL metrics were similar for individuals at different timepoints from the time of surgery over the first year.

Development and implementation of solutions that focus on reducing leakage and improving peristomal skin health may offer potential approaches to improve the quality of life and mental well-being of individuals living with a stoma.

## 2. Introduction

Approximately 13,500–21,000 people in the United Kingdom (UK) undergo stoma surgery each year [1,2]. Stoma formation often exposes patients to various complications, either directly related to the stoma (e.g., high output, dehydration, stomal retraction, necrosis and prolapse) [3] or associated complications, such as stoma-related leakage, peristomal skin complications (PSCs) and reduced psychological well-being [4,5].

Multiple studies have reported on the prevalence of complications related to the stoma (e.g., high output, stoma retraction, necrosis and prolapse) and PSCs within the first period following stoma surgery [6,7,8,9,10,11]. Reports have shown that approximately 15–50% of those with newly formed stomas struggle with PSCs [6,8,9,10]. These reports, however, often rely on retrospective clinical coding data and focus on those stoma-related complications that require admission or prescribed treatment. More general patient-reported-outcome-measures (PROMs) such as leakage, psychological well-being, social engagement and skin-related complications are by comparison under-reported and difficult to measure reliably retrospectively. Previous reports of patient experiences within the first- year following stoma surgery have often relied on qualitative data, especially for the psychological implications of complications associated with living with a stoma [12,13,14]. One quantitative study reported on stoma leakage within the first year, which showed that up to a third of patients reported struggling with day- or night-time leakages; however, this study did not include any recordings of psychological implications in this patient group [11]. Few studies have investigated the psychosocial impact of stoma formation on broader QoL [15,16,17], but these studies did not focus on the implications of leakage, PSCs and other complications and how they may affect QoL.

It is well established for the general stoma population that having a stoma can negatively impact health-related quality of life (QoL) [5,18,19], and the importance of stoma function, and self-efficacy have been demonstrated to be closely associated to QoL [20]. One multinational, cross-sectional study showed that people within one year of stoma surgery were generally more negatively impacted emotionally and socially by their stoma, than people who had their stoma surgery more than a year ago [5].

This study aimed to investigate leakage- and peristomal skin-related complications in those within the first year following stoma formation surgery in the UK. The primary objective of the study was to examine the pattern and degree to which people with a stoma experience stoma-related leakage. Secondary objectives were to assess the wider impact on QoL using stoma-specific- and generic QoL-measurement instruments, patients’ capability of self-management, the prevalence and severity of PSCs and the impact of stoma-related leakage on the ability to work and sleep during the first year following surgery.

## 3. Methods

### 3.1. Study Design

The investigation was a cross-sectional, multi-center study conducted at 3 hospital sites in the UK (The Newcastle Upon Tyne Hospitals NHS Foundation Trust, Lancashire Teaching Hospitals NHS Trust Royal Preston Hospital and James Paget University Hospitals NHS Foundation Trust), evaluating the burden of complications in those who had stoma surgery within the preceding year via a one-to-one consultation with a study nurse (either remotely or face-to-face) and an online, self-reported questionnaire.

Participants were invited for an information- and inclusion visit (V0) and signed consent forms before formally entering the study. Patients who agreed to participate were then invited to a nurse-led interview (V1), which captured baseline demographics and assessed discoloration areas of the peristomal skin. Patients subsequently completed an online questionnaire within 48 h of the nurse-led interview.

### 3.2. Selection of Study Participants

At the respective hospital sites, patients were identified by (1) a screening of hospital surgery databases and patient journals, (2) utilizing a national patient database (Coloplast Charter) and (3) social media recruitment campaigns were produced and posted by the National Institute for Health and Care Research (NIHR) Patient Recruitment Centre: Newcastle. Hospital sites recruited individual patients independently from the sponsor by convenience sampling. 

Since this study also served the purpose of confirming baseline findings in a previous interventional study [21], the following in- and exclusion criteria were established to identify a similar group of patients. Inclusion criteria identified those with an ileostomy or colostomy, being over the age of 18 years and having liquid to mushy effluent (Bristol scale 5–7) [22]. Patients should have had their stoma for less than 12 months and have been self-managing their stoma care products for at least 14 days. Patients should be able to complete an online questionnaire. Patients could not be enrolled if they had limited life expectancy or stage 4 cancer. Patients with a complicated stoma (dehiscence/prolapse/hernia), with more than one abdominal stoma simultaneously, with non-healed abdominal wounds, or with imminent stoma reversal planned prior to the first study visit were excluded. Patients already in the midst of treatment utilizing topical peristomal or systemic steroid treatments were also excluded. Patients who had been enrolled into interventional stoma device trials within the last 12 months were excluded from participation in the current study.

### 3.3. Patient Demographics and Endpoints

Patient demographics and pertinent clinical data (sex, age, stoma type, time since stoma surgery, reason for stoma surgery work status, peristomal body profile and pouching system characteristics) and area of peristomal skin discoloration (using the validated Ostomy Skin Tool v. 2.0 [23,24], which is further described below) were recorded at the nurse-led interview. The remaining endpoints were captured via the self-reported, online questionnaire.

The primary endpoint of this study was the number of times with stoma effluent leakage outside the baseplate (e.g., onto clothes or bed sheets) within the last two weeks.

Multiple secondary endpoints were collected in this study:

(1) Patient self-management using the 13-item version of the Patient Activation Measure (PAM-13) instrument. The PAM-13 instrument is scored on a scale ranging from 0 to 100. Individuals scoring high on this instrument typically understand the importance of taking a proactive role in managing their health and have the skills and confidence to do so [25]. Based on the score, subjects can be grouped into four levels of activation [26]: *Level 1* (Score: 0.0–47.0) indicates that the person does not yet understand his/her role in healthcare. *Level 2* (Score: 47.1–55.1) indicates that the person does not yet have the knowledge and confidence to act. *Level 3* (Score: 55.2–72.4) indicates that the person is beginning to engage in positive health behaviors. *Level 4* (Score: 72.5–100.0) indicates that the person is proactive and engaged in recommended health behaviors.

(2) Burden of leakage using the Ostomy Leak Impact (OLI) tool [27]. The OLI tool consists of 22 questions, which summarizes the burden of leakage in three domains: “*Emotional impact*”, “*Usual and social activities*” and “*Coping and control*”. Each domain sums into a score ranging from 0 to 100, with higher scores reflecting lower impact. The OLI tool has been developed and validated by Coloplast A/S, with the following intraclass correlation coefficients (ICC) for test–retest reliability being reported: “*Emotional impact*” (ICC: 0.885), “*Usual and social activities*” (ICC: 0.814), and “*Coping and control*” (ICC: 0.582) [27].

(3) Health-Related QoL (HRQoL) by the EQ-5D-5L with translation of health-states and index scores being based on the specific value-set for the UK. The second part of the questionnaire consists of a Visual Analogue Scale (EQ-VAS) on which the patient rates their perceived health from 0 (worst imaginable health) to 100 (best imaginable health) [28,29].

(4) Mental well-being using the WHO-5 questionnaire, which is reported on a scale ranging from 0 (worst level of mental well-being) to 100 (highest level of mental well-being) [30].

(5) Peristomal skin health using the Ostomy Skin Tool v. 2.0 [23,24]. The Ostomy Skin Tool v. 2.0 captures both visual and sensory (pain, itching and burning sensations) symptoms of PSCs to better assess the severity for patients. The Ostomy Skin Tool v. 2.0 grades peristomal skin by a decision tree (DT) score ranging from 0 to 3, where a DT score of 0 means no PSC, a DT score of 1 means mild PSC, a DT score of 2 means moderate PSC and a DT score of 3 means severe PSC. The Ostomy Skin Tool v. 2.0 has been developed and validated by Coloplast A/S, with the following ICCs for the tool being reported ICC: 0.735–0.823 [24].

(6) Several additional questions were related to worry about leakage:

Q: *To which degree did you worry about leakage (consider the previous two weeks)?* Response options were on a five-point Likert scale [Very high degree, high degree, some degree, low degree or very low degree/not all at].

Q: *Why did you worry about leakage?* Response options [Odor, embarrassment, soiling of clothes/bed sheets, skin issues, pain, other, I do not worry about leakage].

Q: *What do you normally do when you worry about leakage?* Response options [I live with it/accept it, I use stoma care accessories, I change my pouching system more often, I try another stoma pouching system, I seek advice from my stoma care nurse, I seek advice from peers/other people with a stoma, I seek advice from the manufacturer, I seek advice online, Other, Do not know].

Q: *To what degree has leakage or worry about leakage impacted your sleep (consider the previous two weeks)?* Response options were on a five-point Likert scale [Very high degree, high degree, some degree, low degree or very low degree/not all at].

(7) Additional questions were related to healthcare resource utilization:

Q: *On average how often did you change your baseplate (consider the previous two weeks)?*

Q: *How many times have you changed the baseplate due to worry about leakage (consider the previous two weeks)?*

(8) Finally, few assessments were collected as part of the study:

Q: *How many sick days have you had in the last month?*

Q: *How many sick days have you had due to leakage or worry of leakage in the last month?*

### 3.4. Statistics

The main objective of this study was to investigate and report the prevalence of complications experienced by people living with a newly formed stoma. To fulfil this objective, it was assumed that enrolment of approximately 120–200 participants would be adequate to provide descriptive statistics of complications experienced by this patient group. The study aimed to recruit a similar number of patients in each of the four groups defined by time since surgery (0–3 months; >3–6 months; >6–9 months; >9–12 months) to allow exploratory comparisons of outcome metrics across time since surgery. No formal sample size calculation has been performed due to the exploratory nature of this study. The full analysis set constituted all participants with valid informed consent and with information on at least either the primary or a secondary endpoint.

Endpoints were summarized by descriptive statistics. Continuous variables were summarized with standard metrics (average and standard deviation), and categorical variables were summarized by frequency counts.

Standard least squares models were performed as post-hoc analyses to investigate factors that influence QoL-related metrics (EQ-5D-5L, Visual Analogue Scale and WHO-5). Factors assessed in the respective models were stoma product type (1-piece or 2-piece), stoma product shape (flat, convex or concave), age, body mass index, time since stoma surgery, stoma type (ileostomy or colostomy), gender, skin health (using the decision tree score and assumed to be a continuous variable on a scale from 0 to 3) and number of leakages outside the baseplate in the preceding two weeks. The models were reduced by removing non-significant factors (identified by *p*-values above 0.05).

Additionally, one-way analysis of variance (ANOVA) tests were performed followed by Dunnett’s multiple comparison tests to analyze the relationship between the number of leakage incidents (grouped in categories of 0, 1 or ≥2 leakages in the preceding two weeks) and QoL metrics. All statistical analyses and summaries were made with JMP^®^ v13.1.0 (SAS Institute Inc., Cary, NC, USA) or Graphpad Prism (v9.2.0 for Windows). *p* < 0.05 (*), *p* < 0.01 (**), *p* < 0.001 (***) and *p* < 0.0001 (****).

### 3.5. Ethical Consideration

The study was carried out in accordance with the Declaration of Helsinki and was approved by *London—Bloomsbury Research Ethics Committee* in UK before study initiation (IRAS Project-ID: 301896). The study was registered on ISCTRN (23080097). All participants were fully informed about the investigation, both verbally and in writing, and all gave written informed consent to participate in the study. Participation in the study was voluntary and participants could withdraw from the study at any time. The study was conducted from March 2022 to June 2023 in the UK.

### 3.6. Role of the Funding Source

The study was funded by Coloplast Ltd. The sponsor was involved in the study design, in the analysis and interpretation of data, in writing the report and in the decision to submit the paper for publication.

## 4. Results

### 4.1. Demographics

A total of n = 117 patients were enrolled in the study (provided informed consent). Study enrolment was terminated below the lower target of 120 patients, due to slower recruitment than expected. Three of the enrolled patients did not fulfil all inclusion or exclusion criteria and were hence excluded from the full analysis set (n = 114). One patient failed screening due to having a complicated stoma (dehiscence/prolapse/hernia), one due to not having self-managed stoma appliances for at least 14 days and one due to receiving topical steroid treatment in the peristomal skin area or systemic steroid treatment in the past month. One patient had data recorded at day 366 since stoma surgery; however, this data was allowed by the investigational team to be included in the full analysis set. Patients were enrolled from three hospital sites in the UK (*The Newcastle Upon Tyne Hospitals NHS Foundation Trust*: n = 72, *Lancashire Teaching Hospitals NHS Trust (Royal Preston Hospital)*: n = 32, *James Paget University Hospitals NHS Foundation Trust*: n = 10).

The average age of the participants was 55.8 years (range 18–87; SD = 16.5) and 58% were male. Eighty-eight percent had an ileostomy and 12% had a colostomy. Reasons for stoma formation were cancer (50%), ulcerative colitis (15%) or Crohn’s disease (13%), and the remaining due to other causes (Table 1). On average, participants had their stoma surgery 159.9 days (median 113.5 days) and were discharged 148.1 days (median 103.5 days) prior to completion of the survey (Table 1).

Most participants used 1-piece pouching systems (97%), and participants used baseplates with either convex (55%), flat (38%) or concave (7%) shapes (Table 1). Participants changed baseplate either twice a day or more often (9%), once per day (30%), every second day (49%), or less than every second day (12%).

### 4.2. Leakage Incidents and Worry About Leakage

Participants had on average experienced 1.3 (range 0–14; median = 0) episodes of leakage outside the baseplate in the preceding two weeks (Table 2). More than half the participants (57%) had not experienced any leakage incidents outside the baseplate in the preceding two weeks, 18% had experienced a single leakage incident and a quarter of the participants had experienced two or more episodes of leakage in this period. As many as 74% of participants worried about leakage, 15% to a very high degree (Table 2). The main causes for worrying about leakage were fear of *soiling clothes or bed sheets* (61%), *embarrassment* (53%), *odor* (46%), and *skin issues* (30%). Thirty-nine percent of the participants reported having simply accepted to live with their worry about leakage. A high proportion of the participants addressed their worry about leakage by using stoma care accessories (30%), changing the pouching system more often (39%) or seeking advice from their stoma care nurse (39%). Lastly, participants reported an average of 2.2 baseplate changes per two weeks due to worry about leakage and not as a consequence of actual leakage events (Table 2).

### 4.3. Impact of Leakage on Absence from Work and Sleep

Twenty-nine percent of the participants were employed full-time after their stoma surgery, 38% were retired, 23% were currently on sick leave, 4% were students and 6% were unemployed. The participants that were working full time on average reported having 2.5 (range 0–21; median = 0) sick days in the previous month, of which 0.7 (range 0–14; median = 0) days were attributed to leakage or worry hereof. Leakage or worry regarding leakage impacted approximately two out of three participants in their ability to sleep in the past 14 days, with a quarter of participants being impacted to a *high* (13%) or *very high* degree (11%) (Table 2).

### 4.4. Peristomal Skin Complications

Assessment of the peristomal skin revealed that only 15% of the participants did not have a PSC. Thirty-two percent of the participants had “*severe*” PSCs, 18% had “*moderate*” PSCs and 35% had “*mild*” PSCs. The average decision tree (DT) score was 1.7 (SD = 1.1) for the participants. 

### 4.5. Quality of Life

The average EQ-5D-5L index score was 0.737 (range −0.05–1.00; SD = 0.231), the average EQ-VAS score was 70.2 (range 8–100; SD = 20.0) and the average WHO-5 mental well-being score was 57.9 (range 0–96; SD = 22.8). For the Ostomy Leak Impact tool, the average “*Emotional impact*” domain score was 62.8 (range 3–100; SD = 27.2), the average “*Usual and Social Activities*” domain score was 80.6 (range 4–100; SD = 24.4) and the average “*Coping and in Control*” domain score was 64.7 (range 0–100; SD = 28.8).

### 4.6. Patient Activation

Seven percent of participants were in PAM-Level 1, 24% of participants were in PAM-Level 2, 40% of participants were in PAM-Level 3 and 29% of participants were in PAM-Level 4. The average PAM score was 64.9 (range 38–100; SD = 14.9) for participants of this study.

### 4.7. Associations Between Leakage, PSC, Demographics and QoL Metrics

Standard least squares models were used to assess if there were significant associations between different variables (including number of leakage incidents in the preceding two weeks, decision tree scores (severity of PSCs) and patient demographics (stoma product type, stoma product shape, age, BMI, time since stoma surgery, stoma type, gender)) on the three generic QoL-metrics, being EQ-5D-5L index, EQ-VAS and WHO-5 mental well-being.

These analyses revealed that experiencing leakage(s) outside the baseplate in the preceding two weeks was associated with a significant reduction in scores obtained across all three generic QoL-related metrics. Deterioration of skin condition was associated with significant reductions in EQ-5D-5L index and WHO-5 mental well-being scores, but not in EQ-VAS. Moreover, males had significantly higher EQ-5D-5L index scores than females, and age was positively associated with higher WHO-5 scores (Table 3).

Subsequent analyses were conducted to display associations between leakage incidents and the three generic QoL-metrics, as well as for the three component domains of the Ostomy Leak Impact tool, evaluating participants within groups of those having no leakage, those reporting a single leakage and those suffering from multiple leakages (≥2) within the preceding two weeks.

**Figure 1:**
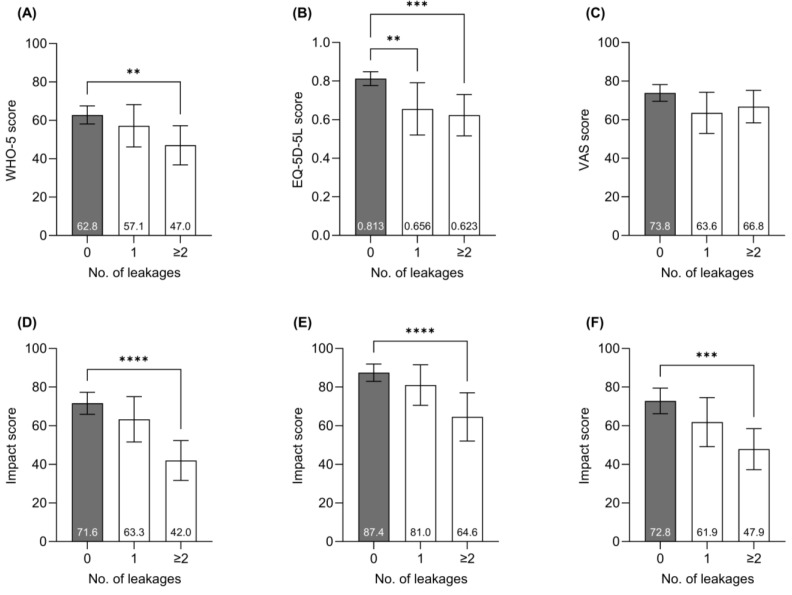
Influence of leakage on QoL-metrics. (**A**) WHO-5 mental well-being score, (**B**) EQ-5D-5L index score, (**C**) EQ-VAS score, (**D**) “*Emotional impact*” domain of OLI tool, (**E**) “*Usual and Social activities*” domain of OLI tool, and (**F**) “*Coping and in Control*” domain of OLI tool. Bars represent average outcome scores, and the error bars represent 95% confidence intervals. One-way ANOVA followed by Dunnett’s multiple comparison tests were performed using the group with no leakages outside the baseplate as reference groups. *p* < 0.01 (**), *p* < 0.001 (***) and *p* < 0.0001 (****).

Participants experiencing ≥two leakage incidents in the preceding two weeks had a significantly lower average WHO-5 score (47.0) than people who had not experienced any leakage incidents (62.8, *p* < 0.01) (Figure 1A). Participants who did not report any leakage incidents in the preceding two weeks had a higher average EQ-5D-5L index score (0.813) than participants who had experienced a single leakage incident (0.656, *p* < 0.01)) or ≥two incidents (0.623, *p* < 0.001) (Figure 1B). No significant difference in EQ-VAS scores was observed between leakage groups (Figure 1C), but having multiple leakage incidents was associated with significantly worse outcomes in all three domains of the OLI tool (Figure 1D–F).

### 4.8. Time Since Surgery

It was further assessed if the time since stoma surgery was associated with differences in patients’ outcomes. No significant changes in baseline values were observed as a function of time since stoma surgery for any of the QoL-related outcomes assessed. Nor was the time since surgery associated with any significant differences in the number of leakage incidents, skin health (decision tree score) or patient activation (PAM-13) (Table 4).

## 5. Discussion

This study sought to characterize the impact of living with a stoma in a population of patients who had their stoma formed within the preceding year, and reports on the prevalence of stoma-related complications, the impact of these on their daily lives and healthcare resource utilization. The principal findings describe that people with a new intestinal stoma experience significant leakage and PSC complications, as well as negative impact on QoL, but concerningly none of the QoL outcome metrics improved significantly throughout the first year following stoma surgery.

Almost half the participants (43%) had experienced one or more episodes of feces leaking outside the baseplate in the preceding two weeks, which corroborates other studies showing that many people with a newly formed stoma, as well as experienced users, struggle with leakage incidents both at day and during night-time [5,8,31,32,33]. A large multinational study showed that the proportion of people experiencing weekly episodes of stoma leakage was higher among people with a newly formed stoma (surgery within the preceding year) than people who had been living with their stoma for more than a year [5], indicating that it may take time to optimize product selection and adopt proper stoma care routines to mitigate the risks of leakage. In line with this, one-third of the participants in this study were in PAM levels 1 and 2, indicating that they were struggling with their self-care and healthcare management throughout the first year, which could contribute to a negative impact on their stoma care.

Leakage incidents are inconvenient and are reported to have profound negative psychosocial consequences for the afflicted [5,34,35,36,37]. In the present study focusing on individuals with a new stoma, leakage was also associated with reductions in all three generic QoL-metrics (WHO-5, EQ-5D-5L and EQ-VAS). Participants struggling with leakage scored significantly lower on the WHO-5 mental well-being index, with the score decreasing Δ15.8 points, from 62.8 for participants with no leakage incidents to 47.0 for participants with ≥two leakage incidents in the preceding two weeks. A 10-point difference on this scale is considered clinically relevant and people scoring below 50 are considered at risk of depression [30,38]. Participants with no leakage incidents in the preceding two weeks had WHO-5 mental well-being scores that were similar to the general UK population (the average level in 2016 was 60.7 in those aged 50–64 [39]). The EQ-5D-5L instrument was used to assess participants’ wider health-related QoL. The average EQ-5D-5L index score in the present study was 0.737 (UK specific), which is lower than that reported for the general population in England (0.885) [40]. Participants who did not experience any episodes of leakage in the preceding two weeks had an average index score of 0.813, whilst people with ≥two leakage incidents in the preceding two weeks had an average index score of 0.623. These scores are similar to that reported from a time trade-off study describing scores for people with a stoma experiencing no leakage and four leakage incidents per month, respectively [41]. The average EQ-VAS score was 70.2 in this trial, thus, much lower than the average self-rated VAS score of 80 (female) and 84 (male) reported for the general population aged 55–64 years in the UK [42].

For the specific Ostomy Leakage Impact tool, participants scored significantly lower in all three domains of the OLI tool when experiencing ≥two leakage incidents in the preceding two weeks compared to people who had not experienced any leakage incidents. A previous study, which included experienced individuals and individuals with a newly formed stoma, showed that a single leakage incident within the preceding year was associated with a significant reduction in the “Emotional Impact” domain score [5]. In our study, participants experiencing a single leakage incident in the previous two weeks reported scores trending lower, yet insignificantly, in all three OLI domains. The reason for this discrepancy is probably due to the relatively low number of participants reporting a single leakage incident in this study (n = 18) versus the referenced study that had >3000 participants in the analysis [5]. These data indicate that people within the first year after stoma formation in the UK experience lower health-related QoL and mental well-being compared with the general population and that the degree to which people struggle with leakage is associated with a reduction in the different QoL-related metrics. Solutions that can reduce stoma leakage incidents have previously been correlated with improvements in QoL, examples include studies on the selection of optimal pouching systems for individuals’ peristomal body profiles ensuring a tight seal between baseplate and peristomal skin [43], or the outcomes of stoma care products that can reduce the risk of leakage [21,33,44,45,46].

In the present study, 85% of the participants had PSCs ranging from mild to severe, which is generally higher than the prevalence of PSCs described in the literature, which has previously been reported at approximately 15–50% in those with newly formed stomas [6,8,9,10]. The reported prevalence in the present study is, however, similar to that reported in a large multinational cross-sectional study for the general stoma population using the same peristomal skin assessment tool (Ostomy Skin Tool 2.0) [23]. The discrepancies in reported prevalences of PSCs may be due to a dependence on less accurate or sensitive hospital coding episodes in some studies and the variance in type and sensitivity of the tools used to describe peristomal skin health [23,47,48,49]. This supports the rationale for undertaking this cross-sectional study on the basis that PSCs may be underreported if reliant on coding data. In the present study, we used the validated Ostomy Skin Tool 2.0, which captures both visual signs and non-visual symptoms (pain, itching and burning sensations) of PSCs to better assess the severity of symptoms [23,24], compared with other assessment tools that predominantly capture visual signs of PSCs [48,49]. Health-related QoL (assessed with EQ-5D-5L) and mental well-being (assessed with WHO-5) were reduced for people with PSCs in the present study, corroborating previous observations that living with PSCs in the general stoma population is associated with negative implications on QoL as well [34,41,50,51,52]. Future lines of innovation and development aimed at reducing PSCs may contribute to wider effects including improved QoL. However, the risk of experiencing PSCs may not necessarily be solved with product innovations alone, but will also rely on the development and implementation of educational interventions directed at both patients and health professionals to establish best practices in the stoma care routine to minimize the risk of getting PSCs [53,54]. The age of participants was positively associated with mental well-being (WHO-5) and gender influenced the EQ-5D-5L index score, with males having significantly higher EQ-5D-5L index scores than females. Similar findings have been reported of males scoring marginally, but significantly higher than females on the VAS instrument, and that age was positively associated with the “City of Hope National Medical Center Quality of Life Questionnaire” for patients with a stoma [34]. However, multiple studies have been conducted reporting on associations between age and gender on various QoL-metrics for people living with a stoma, with the implications of age and gender differing from relevant to irrelevant between studies [17,18,55,56,57]. Identification of high-risk individuals with low QoL early after stoma surgery is an interesting future line of research.

On the other hand, none of the QoL-related outcomes (OLI domains, WHO-5, EQ-5D-5L index score or EQ-VAS) assessed in the present study were significantly different between individuals at different time points within the first year following stoma surgery, corroborating previous findings for a similar cohort of patients [21] and being in line with a recent longitudinal study showing marginal, but insignificant, improvement in QoL within the first year following stoma surgery [17]. Two other longitudinal studies assessing the short-term impact of stoma surgery on QoL (assessed with the generic SF-36 vs. 2.0 and the cancer-patient specific EORTC-QLQ-C30) indicated that people were most negatively impacted right after surgery (one–two months post-surgery), with an observed QoL-score improvement in the following months, before reaching a plateau with more stable QoL metrics [15,16]. However, we did not observe lower QoL for participants reporting immediately after stoma surgery. This discrepancy may be due to differences in stoma support for the current population, the choice of QoL-assessment tool or differences in the collection of data (cross-sectional data collected after stoma surgery vs. longitudinal data with data both pre- and post-surgery). These findings are, however, a cause for concern and further research is needed to investigate why participants who had been living longer (up to a year) with their stoma did not report better in the assessed QoL outcomes than participants with shorter experiences of living with a stoma. The present study did not collect data on the type and amount of pre- and post-operative education of patients within stoma care and their general access to stoma care nurses in the year following stoma formation, which are fundamentally important for patients to establish good routines and thus minimize the risk of experiencing complications associated with living with a stoma [58]. Future research should include these aspects to establish if changes are needed in the provision of patient education and follow-up interactions throughout the first year of living with a stoma to ensure that patients receive optimal care to minimize the risks of experiencing complications.

Living with a stoma may not only have a negative psychosocial impact on the individual but can also have an impact on the utilization of healthcare resources, especially amongst those who struggle with leakages [5,32]. These previous reports have shown an increased use of stoma care accessories in patients who worry about leakage [5] and that people struggling with leakage both use more stoma care products (pouching systems and accessories) and interact more often with health professionals [32]. The current study supports that worry about leakage is associated with the use of additional stoma care products, either by increased frequency of changing the pouching system or by using additional accessories.

Despite advances in care for people with a stoma, stoma-related complications remain high throughout the first year post-surgery and impose increased utilization of healthcare resources, and also affect people’s ability to work [59,60]. Treatment of complications associated with living with a stoma, such as PSCs [61,62,63,64] and stoma-leakage [32], have a considerable impact on the use of healthcare resources and the associated costs on an individual level, but also impact at a socioeconomic level in affecting return to work. In the present study, those employed reported having an average of 0.7 sick days per month due to leakage-related issues or worries regarding it. However, these numbers were driven by a few participants reporting the majority of sick days in the preceding month due to leakage-related issues or worries regarding it. Other studies have likewise shown that stoma formation and the required management and care can have a negative impact on the ability to work, potentially forcing a change in occupation or leading to periods of sickness absence or early retirement [5,59,65,66,67]. More thorough investigations are needed to elucidate the implications of stoma-related complications on this important topic.

### Limitations

The insights presented in this report should be interpreted whilst considering several study limitations. The cross-sectional study design only allows the determination of associations between collected variables and outcomes and does not warrant conclusions on causal relationships between such variables and outcomes. The study is limited by the variables collected in the study and, thus, we cannot exclude that other variables not collected as part of this study, such as access to stoma care nurses, pre- and post-surgery stoma management education etc., can have an impact on QoL following stoma formation. Furthermore, the study was not designed and powered to investigate specific associations between variables. No formal sample size calculation has been conducted and the analyses on associations between variables were not pre-specified in the statistical analysis plan and, therefore, these analyses are to be considered exploratory in nature.

Only patients with liquid to mushy effluent (Bristol scale 5–7) [22] were included in this study. This inevitably leads to an increased proportion of ileostomy patients given the nature of small bowel effluent. People with an ileostomy often have a higher risk of experiencing leakages and PSCs than people with a colostomy, due to more liquid and corrosive effluent [5,68,69,70] and this may also in part explain the higher rates of PSCs in this study. People with non-healed abdominal wounds, those with advanced cancer and those with more than one stoma were excluded from the study and, therefore, we may under-report the proportion of people struggling with severe PSCs within these higher-risk groups. With the inclusion and exclusion criteria for study participation, the results are not necessarily representative of the general newly discharged stoma population in the UK, and the fact that the study was only conducted in the UK may also limit generalizability to the international stoma population.

## 6. Conclusions

The data reported herein suggest significant levels of complications being experienced by individuals within the first year of stoma surgery, including significant problems with leakage and PSCs, which are debilitating for the afflicted, and costly to the healthcare system. Leakage and PSCs were associated with lower QoL in patients with a newly formed stoma, corroborating previous evidence on the general stoma population. Concerningly the QoL scores were similar for individuals at different timepoints from the time of surgery over the first year. Our study warrants further research to investigate and understand why participants who had been living longer with their stoma did not report better in the assessed QoL outcomes than participants with shorter experiences of living with a stoma. Interventions specifically focused on addressing leakages, worry about leakage and PSCs could contribute to better QoL, with potential beneficial effects on healthcare resource utilization and return to employment.

## Figures and Tables

**Table 1:** Demographics of intention-to-treat population. BMI = Body Mass Index.

Parameter	Total (n = 114)
Age (years): Mean ± SD (range)	55.8 ± 16.5 (18; 87)
Sex: n (%)	
Female	48 (42%)
Male	66 (58%)
Days since stoma surgery to V1: Mean ± SD (range)	159.9 ± 110.1 (23; 366)
Days since discharge from hospital to V1: Mean ± SD (range)	148.1 ± 110.9 (1; 359)
Type of stoma: n (%)	
Ileostomy	100 (88%)
Colostomy	14 (12%)
Reason for stoma creation: n (%)	
Ulcerative colitis	17 (15%)
Cancer	57 (50%)
Crohn’s Disease	15 (13%)
Other	25 (22%)
Body profile: n (%)	
Inward	16 (14%)
Outward	55 (48%)
Regular	43 (38%)
Pouching system type	
1-piece	111 (97%)
2-piece	3 (3%)
Baseplate shape	
Flat	43 (38%)
Convex	63 (55%)
Concave	8 (7%)
BMI (kg/m^2^): Mean ± SD (range)	26.8 ± 5.7 (16; 47)

**Table 2:** Leakage incidents, worry about leakage and impact on sleep.

Parameter	Total (n = 114)
**Number of leakage episodes outside baseplate per two weeks**: Mean ± SD (range)	1.3 ± 2.5 (0; 14)
**To which degree did you worry about leakage (consider the previous two weeks)?** n (%)	
Very high degree	17 (15%)
High degree	13 (11%)
Some degree	33 (29%)
Low degree	21 (18%)
Very low degree/not at all	30 (26%)
**Why did you worry about leakage?** n (%) *	
Odor	53 (46%)
Embarrassment	60 (53%)
Soiling of clothes or bed sheets	69 (61%)
Skin issues	34 (30%)
Pain	10 (9%)
Other	3 (3%)
I do not worry about leakage	21 (18%)
**What do you normally do when you worry about leakage?** n (%) *	
I live with it/accept it	44 (39%)
I use stoma care accessories	34 (30%)
I change my pouching system more often	45 (39%)
I try another stoma pouching system	12 (11%)
I seek advice from my stoma care nurse	44 (39%)
I seek advice from peers/other people with a stoma	7 (6%)
I seek advice from the manufacturer	1 (1%)
I seek advice online	15 (13%)
Other	3 (3%)
Do not know	1 (1%)
**To what degree has leakage or worry about leakage impacted your sleep (consider the previous two weeks)?** n (%)	
Very high degree	13 (11%)
High degree	15 (13%)
Some degree	19 (17%)
Low degree	25 (22%)
Very low degree/not at all	42 (37%)
**How many times have you changed the baseplate due to worry about leakage in the last two weeks?** Mean ± SD (range)	2.2 ± 3.2 (0; 14)

* More than one response option could be chosen.

**Table 3:** Key variables influencing generic quality of life metrics. Non-significant factors were removed from the respective models (*p* > 0.05). Parameter estimates are per unit change (*Leakage episode outside baseplate* (per number on a scale ≥ 0), *Decision tree score* (per number on a scale from 0 to 3) and *Age* (per year)).

Metric	Variables	Parameter Estimate	95% CI	*p*-Value
EQ-5D-5L	Leakage outside baseplate	−0.0215	−0.0378 to −0.0052	0.0103
	Decision tree score	−0.0478	−0.0856 to −0.0099	0.0140
	Gender			
	[Female]	−0.0452	−0.0859 to −0.0046	0.0295
	[Male]	0.0452	0.0046 to 0.0859	0.0295
EQ-VAS	Leakage outside baseplate	−1.81	−3.27 to −0.35	0.0158
WHO-5	Leakage outside baseplate	−2.65	−4.18 to −1.11	0.0009
	Decision tree score	−5.44	−9.03 to −1.85	0.0033
	Age	0.33	0.11 to 0.56	0.0052

**Table 4:** Impact of time since surgery on baseline values of different outcome measures.

Parameter	Slope (Score Change/Day)	*p* Value
Leakage outside baseplate	0.000195	0.928
PAM-13 score	0.00122	0.924
Decision tree score	−0.00024	0.799
Emotional impact (OLI)	−0.02145	0.358
Usual and social activities (OLI)	0.007478	0.729
Coping and control (OLI)	0.010142	0.682
EQ-5D-5L (Index)	−0.00004	0.825
EQ-VAS	−0.00055	0.974
WHO-5	−0.01108	0.572

## Data Availability

Anonymous data, study protocol and informed consent form are available from the corresponding author on reasonable request.

## Public Involvement Statement

No public involvement in any aspect of this research.

## Guidelines and Standards Statement

This manuscript was drafted according to the STROBE Statement for cross-sectional studies.
